# Supplementary material for: Pathways of TB Transmission in Children—A Systematic Review of Molecular Epidemiological Studies
Source: Int J Environ Res Public Health. 2023 Jan 18;20(3):1737. doi: 10.3390/ijerph20031737 (PMC9914148; doi:10.3390/ijerph20031737)
Supplement: Supplementary file 1 [file ijerph-20-01737-s001.zip › ijerph-2081855-supplementary.pdf]

Supplementary Table S1.

Study (first author, year)	Country	Observation Time	Study Type	Type of Genotyping	Patients with Active TB (tuberculosis) included (no.)	Patients Genotyped (no.)	Culture-Confirmed Children < 15 yr (no.)	Children with Pulmonary TB (no.)
Ahmad S et al., 2003 [14]	Beirut (Lebanon)	1996	Case series	Double-repetitive-element (DRE)-PCR (Polymerase Chain Reaction)	6	6	1	1
Aleinikova D et al., 2020 [15]	Latvia	2016	Case study	WGS (whole-genome sequencing)	2	2	1	1
Andre M et al., 2007 [16]	US (Oklahoma)	August 2001 and December 2002,	Outbreak analysis	Spoligotyping, IS6110-RFLP (restriction fragment polymorphism)	38	14	10	n.a.
Augustynowicz-Kopeć E et al., 2012 [17]	Poland	1/2003 to 7/2010	Cross-sectional	Spoligotyping, MIRU-VNTR (Mycobacterial interspersed repetitive unit-variable number tandem repeat)	78	78	2	2
Bang D et al., 2010 [18]	Denmark	1992–2007	Retrospective nationwide cross-sectional study	Spoligotyping, IS6110-RFLP	29	29	3	n.a.

Bishara H et al., 2014 [19]	Israel	May 2013–January 2014	Outbreak analysis	Spoligotyping MIRU-VNTR	11	6	1	0 (TB meningitis)
Buff AM et al., 2010 [20]	South Carolina (US)	January 2005 to December 2007	Outbreak Analysis	MIRU-VNTR	21	13	5	4
Chin DP et al., 2000 [21]	San Francisco Area (US)	1996–1997	Population-based	IS 6110-RFLP, PGRS (polymorphic guanine-cytosine rich-sequence)	73	73	19	n.a.
Comin J et al., 2020 [22]	Aragon (Spain)	2014–2017	Outbreak analysis	Spoligotyping, MIRU-VNTR	14	14	1	0 (peritoneal TB)
Cronin WA et al., 2002 [23]	Maryland (US)	1996–2000	Cross-sectional	Spoligotyping, IS6110-RFLP	1154	1172	14	n.a.
Curtis AB et al., 1999 [24]	North Dakota (US)	1998	Outbreak analyis	IS 6110-RFLP, PGRS	2	2	1	1 (2)
Dahle UR, et al., 2005 [25]	Norway	February to September 2002	Outbreak analysis	IS 61110-RFLP	15	14	5	5
Dewan PK et al., 2006 [26]	San Francisco (US)	September 2003 to July 2004	Outbreak analysis	IS 6110- RFLP	11	6		4
Duarte R et al., 2009 [27]	Portugal	2007	Case report	MIRU-VNTR, IS6110-RFLP	2	2		1
Ewer K et al., 2003 [28]	UK	February to April 20021	Outbreak analysis	IS 6110-RFLP	69	10	10	10
Faccini M et al., 2013 [29]	Milan (Italy)	November 2010 to February 2011	Outbreak analysis	"Genotyping"	15	11	11	11
Filia A et al., 2011 [30]	Italy	November 2008 to January 2009	Outbreak analysis	"DNA fingerprinting"	19	10		n.a.

Garcia de Viedma D et al., 2005 [31]	Madrid (Spain)	January 2001 to December 2002	Cross sectional	Spoligotyping, IS6110-RFLP	16	16	1	1
Joean O et al., 2020 [32]	Germany	May 2018 and March 2020	Case series	WGS	7	6	3	3
Kohl TA et al., 2014 [33]	Hamburg, Germany	2001 to 2010	Outbreak analysis	Spoligotyping, IS6110-RFLP, cgMLST (core genome multilocus sequence typing)	26	26	2	2
Kozinska M et al., 2016 [34]	Poland	2003–2013	Cross sectional	Spoligotyping, IS 6110-Mtb1-MTb2 PCR, MIRU-VNTR	150	150	8	n.a.
Kozinska M et al., 2022 [35]	Poland	2020	Case report	Spoligotyping, MIRU	2	2	1	1
Kubica T et al., 2004 [36]	Germany	1995–2001	Cross sectional	Spoligotyping, IS 6110-RFLP	451	451	2	n.a.
Kubin M et al., 2001 [37]	South Moravia (Czech Republic)	1993–1999	Outbreak analysis	IS6110-RFLP	184	17	2	n.a.
Kumar K et al., 2013 [38]	UK	n.a.	Outbreak analysis	MIRU-VNTR	7	6		1
Lopez-Calleja AL, et al., 2009 [39]	Zaragoza (Spain)	June 2001–May 2004	Cross-sectional	Spoligotyping, IS6110- RFLP	85	85	11	n.a.

Lowther SA et al., 2008 [40]	Minnesota (US)	January–September 2008	Outbreak analysis	Spoligotyping, MIRU-VNTR	16	6	1	1
Marais B, et al. 2009 [41]	Cape Town (South Africaq)	January 2003–December 2004	Prospective, community-based	IS6110- RFLP	221	164	6	n.a.
McElroy PD et al., 2002 [42]	US (Baltimore, New Jersey, NY)	1996–2001	Outbreak analysis	IS6110- RFLP	39	36	1	n.a.
Mitruka K et al. 2014 [43]	Southern Nevada (US)	May 2006–August 2008	Outbreak analysis	MIRU-VNTR, IS6110- RFLP	8	8	2	2
Monnan PK et al. 2013 [44]	8 States (US)	2007–2009	Cross-sectional	Spoligotype 12-locus MIRU-VNTR	92	92	2	n.a.
Nguyen D et al., 2003 [45]	Nunavik, Quebec, (Canada)	1999–2000	Cross-sectional	IS6110-RFLP, Spoligotyping, MIRU-VNTR	51	48	1	n.a.
Nordholm A et al., 2019 [46]	Denmark	2000–2013	Cross sectional (nationwide register-based)	MIRU-VNTR, IS6110-RFLP	88	34	35	25
Paranjothy S et al. 2008 [47]	UK	n.a.	Outbreak analysis	MIRUVNTR	2			2
Sun SJ et al., 2002 [48]	US (seven sites)	1996–2000	Cross-sectional	Spoligotying, IS6110-RFLP	10752	114	114	91
Tang TQ et al., 2014 [49]	Taiwan	2000–2006	Case report	Spoligotyping, MIRU-VNTR	4	4	2	2

Thomas TA et al, 2014 [50]	Virginia, US	December 2004–August 2010	Outbreak analysis	Spoligotyping, IS6110-RFLP, MIRU-VNTR	30	23	6	n.a.
Van Rie A, et al., 1999 [51]	Cape Town , South Africa	1993–1997	Outbreak analysis	IS6110-RFLP	21	21	1	n.a.
Wootton SH et al, 2005 [52]	Houston (US)	October 1995, to September 2000.	Cross sectional	Spoligotyping, IS 6110-RFLP	177	78	50	n.a.

Supplementary Table S2.

Study (first author, year)	Clusters	Culture-Confirmed Patients in Clusters (no.)	Clusters Including Children (no.)	Children in Clusters (no.)	Contact Tracing Performed?	Transmission to Secondary Cases (no.)	Source Cases of Children	Child Putative Source ?
Ahmad S et al., 2003 [14]	1	2	1	1	no	1	33-y-old father	no
Aleinikova D et al., 2020 [15]	1	2	1	1	yes	1	Father	no
Andre M et al., 2007 [16]	1	13	1	n.a.	yes,	n.a.	23- year-old man	no
Augustynowicz-Kopeć E et al., 2012 [17]	35	78	2	2	yes	43	(1) Father of 2-year-old son; (2) Sputum smear-positive mother diagnosed 2 months later than smear-negative 1-year-old daughter	unclear in (2)
Bang D et al., 2010 [18]	1	5	1	1	n.a.	1	Adult asylum seeker	no
Bishara H et al., 2014 [19]	1	6	1	1	yes	5	22-year-old Israel Arab	no
Buff AM et al., 2010 [20]	1	13	1	5	n.a.	n.a.	Adult household contacts	no
Chin DP et al., 2000 [21]	1	73	1	19	yes	41	n.a.	no (not mentioned)

Comin J et al, 2020 [22]	1	14	1	1	yes	0	Gambian relative	no
Cronin WA et al., 2002 [23]	111	436	n.a.	n.a.	yes	155	not reported	no
Curtis AB et al., 1999 [24]	1	3	1	1	yes	1	9-year-old boy infected 36-year-old female guardian)	yes
Dahle UR, et al., 2005 [25]	1	9	1	3	yes	8	All 9 cluster pts attended the same church; age of source case not mentioned	no
Dewan PK et al., 2006 [26]	1	6	1	4	yes	5	27-year-old man	no
Duarte R et al., 2009 [27]	1	2	1	1	yes	1	Adult woman	no
Ewer K et al., 2003 [28]	1	10	1	10	yes	9	Pupil (school year 9)	yes
Faccini M et al., 2013 [29]	1	3	1	2	yes	2	17-year-old adolescent	no
Filia A et al., 2011 [30]	1	10	1	9	yes	9	42-year-old female assistant	no
Garcia de Viedma D et al., 2005 [31]	2	16	1	1	no	1	Unknown	no

Joean O et al., 2020 [32]	1	6	1	3	yes	5	50-year-old migrant	no
Kohl TA et al., 2014 [33]	1	26	1	2	yes	13	Adult	no
Kozinska M et al., 2016 [34]	18	138	7	8	no	n.a.	Adults; in one family source case unknown	no
Kozinska M et al., 2022 [35]	1	2	1	1	yes	1	Mother	no
Kubica T et al., 2004 [36]	46	433	2	2	n.a.	39	Mother and grandmother	no
Kubin M et al, 2001 [37]	6	17	2	2	yes	n.a.	Father and grandfather	no
Kumar K et al, 2013 [38]	1	6	1	1	yes	5	26-year-old man	no
Lopez-Calleja AL et al., 2009 [39]	1	85	1	11	no	16	Father (n = 2 transmissions); Mother (n = 1 transmission); Nurses (n = 8 transmissions)	no
Lowther SA et al., 2008 [40]	1	4	1	1	yes	3	25-year-old Guatemalan	no
Marais B et al. 2009 [41]	n.a.	n.a.	2	2	yes	2	Adults	no
McElroy PD et al., 2002 [42]	1	34	1	1	no	1	Adult (transgender)	no
Mitruka K et al. 2014 [43]	1	8	1	2	yes	7	Fathers	no

Monnan PK et al. 2013 [44]	14	26	2	2	yes	14	Adult hispanic immigrants	no
Nguyen D et al., 2003 [45]	6	46	1	1	no	17	Young adult	no
Nordholm A et al., 2019 [46]	n.a.	31	n.a.	31	yes	n.a.	Primarily infected by parents	no (not stated)
Paranjothy S et al., 2008 [47]	1	2	1	2	yea	1	9-year-old boy of Black African ethnicity	yes
Sun SJ et al., 2002 [48]	n.a.	88	?	44	yes	n.a.	Mean age of source cases ± 31 years.	no (not stated)
Tang TQ et al., 2014 [49]	1	4	1	2	yes	3	Aboriginal tunnel worker	no
Thomas TA et al., 2014 [50]	1	23	1	1	yes	17	Adults	no
Van Rie A et al., 1999 [51]	1	16	1	1	yes	12	29-year-old mother	no
Wootton SH et al., 2005 [52]	n.a.	51	n.a.	43	no	3	Adults source cases of 3 children (1-,7- and 14- years old); age of other known sources not specified	n.a.
